# International Women’s Day 2014: women’s health equity is progress for all

**DOI:** 10.3332/ecancer.2014.ed33

**Published:** 2014-03-07

**Authors:** Ophira Ginsburg

**Affiliations:** Scientist, Women’s College Research Institute and Assistant Professor, Medicine, Public Health, University of Toronto, Canada

Today is International Women’s Day, a day borne out of the labour and suffrage movements in North America and Europe at the turn of the last century. Since then March 8th has become a day to reflect upon the status of women in our own communities and beyond, to take note of our progress and ongoing challenges, and to honour the many women who contribute to the struggle for women’s equality, whether in villages, cities, or on the international stage. It’s also an opportunity to celebrate the special women in our lives: grandmothers, mothers, daughters, sisters, wives, partners and friends. This year the UN will mark the event on the eve of the 58th session of the Commission on the Status of Women. The theme for IWD 2014 is **“Equality for women is progress for all”** [1]. 

Let us first consider the status of women in the countries in which we live. Are we there yet? What of women’s health in places with limited resources, in low-and middle-income countries (LMICs) of Latin America, Africa and Asia, and in the least developed regions where competing priorities include the basics: clean drinking water, sanitation and food safety? 

And where do women’s cancers fit on the global health agenda? 

In 2012 there were 14.1 million new cases, 8.2 million cancer deaths and 32.6 million people living with cancer [2]. Breast and cervical cancer took the lives of 522,000 and 266,000 women respectively-that’s half a million more women who died from these two cancers alone, than from complications in pregnancy or childbirth [3]. From Bangladesh to Brazil, in low-and middle-income countries on every continent, cancer is now among the top causes of premature death and disability. Recent reports highlight the rapidly increasing global burden of cancer, a so-called “tidal wave” with population aging and human development [4, 5]. In keeping with the “cancer transition” [6], the incidence of breast cancer over the next 10–20 years is expected to be particularly dramatic, as development comes with a predictable shift in reproductive risk factors (i.e. later age at first birth, parity and breastfeeding). Over 85% of cervical cancer deaths occur in less developed regions, where women with breast cancer usually present with advanced disease. For most of the world’s population, access is limited not only to breast and cervical screening but also to safe surgery, radiotherapy, as well as supportive and palliative care [2, 7–8]. 

So where is our outrage? Aside from a few small-scale programs [9], woefully little attention and negligible funding has been raised or diverted towards this looming public health disaster. In keeping with the “health is wealth” paradigm, there is a case to be made for investing in breast and cervical cancer control in LMIC; the opportunity cost of continuing to do nothing was highlighted in the Report of the Global Task for on Expanded Access to Cancer Care and Control (GTF.CCC [10]). And might close to a million women dying unnecessarily each year be viewed through the lens of social justice, of human rights? 

Women’s health should mean more than just reproductive health. In Bangladesh, a mother’s death from any cause greatly affects a child’s survival to age 10 (24% versus 89%, p<0.0001) [11]. While the greatest impact on survival is seen when the mother dies during the neonatal period, there is still a 16% absolute difference in a child’s cumulative probability of survival even if the mother lives past his or her first birthday. Given the young ages at which women develop breast and cervical cancer in low-resource settings, children whose mothers die from these cancers become “cancer orphans” [12]. Without considering the costs of care (if any is available), a diagnosis of breast or cervical cancer thus contributes to the cycle of poverty. Those who do survive cancer risk being abandoned by their families, ostracized by their communities, or worse [13]. Women’s empowerment can therefore be viewed as the basis for and the consequence of effective breast and cervical cancer control. 

On a hopeful note, as the economics case for investing in global cancer control begins to take shape and cancer finally has a place on the global health agenda [14], the time has come to place women’s health equity among the core elements of global cancer policy. It is encouraging to note the recent attention towards access to cervical screening and HPV vaccination in LMIC [15–17]. And efforts to increase “breast awareness” and even CBE can arguably be of value in resource-constrained settings where profound gender inequity, stigma and taboos still exist; regions where the average woman presents with bulky, node positive, often inoperable disease. Capacity-building for safe, good quality surgery and radiation have rarely been mentioned in global health discussions; but this, too may change, as the Lancet Global Surgery Commission and the UICC Global Task Force on Radiotherapy for Cancer Control (GTF.RCC) embark on their challenging work in 2014. 

Finally, we can take heart from the increasing number of women who choose careers in cancer care and research [18]. International Women’s Day can be a time to celebrate and encourage more women to become leaders in oncology: women like Dr Sandra Swain, past-president of ASCO, Dr Martine Piccart, past-president of EORTC and current president of ECCO, Dr Mary Gospodarowicz, medical director of Princess Margaret Cancer Centre and president of UICC, and Dr Felicia Knaul, economist and Director of the Harvard Global Equity Initiative who co-founded and leads the GTF.CCC. 

Perhaps, on this International Women’s Day we can take a moment to reflect on three things:

1. While there remains much to be done to decrease preventable suffering and death from women’s cancers throughout the world, there has been great progress, with the dramatic decreases observed in the incidence of cervical cancer and mortality from breast and cervical cancer in high-income countries. These advances are paralleled by improvements in the status of women, in women’s rights and opportunities.
2. The health of women reflects their status in society, and can be both a driver and consequence of efforts to combat stigma, fears and myths about cancer, and taboos about women’s bodies.
3. Women leaders in oncology can play an important role in improving the dire situation for millions who have little or no access to care, not only in low- and middle-income countries but also in less-resourced, remote, or otherwise marginalized communities in high income countries.

Morbidity and mortality from women’s cancers are shamefully high. Affordable access to early detection and proper treatment for breast and cervical cancer, which disproportionally affects the world’s most disadvantaged women, should be a human rights as well as an economic priority. Equality for women is indeed progress for all. 

## Notes

Watch Professor Martine Piccart and Professor Felicia Knaul on ecancertv.
